# Survival of *Bacillus* spp. SUBB01 at high temperatures and a preliminary assessment of its ability to protect heat-stressed *Escherichia coli* cells

**DOI:** 10.1186/s13104-015-1631-9

**Published:** 2015-11-03

**Authors:** Md. Sakil Munna, Jannatun Tahera, Md. Mohibul Hassan Afrad, Ifra Tun Nur, Rashed Noor

**Affiliations:** Department of Microbiology, Stamford University, 51 Siddeswari Road, Dhaka, 1217 Bangladesh

**Keywords:** High temperature stress, *Bacillus* spp., Colony forming units (CFUs), Viable but non culturable (VBNC) cells

## Abstract

**Background:**

The bacterial stressed state upon temperature raise has widely been observed especially in *Escherichia coli* cells. The current study extended such physiological investigation on *Bacillus* spp. SUBB01 under aeration at 100 rpm on different culture media along with the high temperature exposure at 48, 50, 52, 53 and 54 °C. Bacterial growth was determined through the enumeration of the viable and culturable cells; i.e., cells capable of producing the colony forming units on Luria–Bertani and nutrient agar plates up to 24 h. Microscopic experiments were conducted to scrutinize the successive physiological changes. Suppression of bacterial growth due to the elevated heat was further confirmed by the observation of non-viability through spot tests.

**Results:**

As expected, a quick drop in both cell turbidity and colony forming units (~10^4^) along with spores were observed after 12–24 h of incubation period, when cells were grown at 54 °C in both Luria–Bertani and nutrient broth and agar. The critical temperature (the temperature above which it is no longer possible to survive) of *Bacillus* spp. SUBB01 was estimated to be 53 °C. Furthermore, a positive impact was observed on the inhibited *E. coli* SUBE01 growth at 45 and 47 °C, upon the supplementation of the extracellular fractions of *Bacillus* species into the growing culture.

**Conclusions:**

Overall the present analysis revealed the conversion of the culturable cells into the viable and nonculturable (VBNC) state as a result of heat shock response in *Bacillus* spp. SUBB01 and the cellular adaptation at extremely high temperature.

## Background

*Bacillus* species are well known spore-forming pathogenic bacteria which are frequently found in the environment. Like *Escherchia coli*, these bacterial species may encounter a number of growth retarding stress factors, including nutrient depletion, temperature fluctuation, variation in pH and redox potential, limited water activity (a_w_), elevated level of reactive oxygen species (ROS), osmotic imbalance along with unusual solute concentrations, etc. [1–15]. In response to such stress causing stimuli, different bacterial species have been observed to employ various defensive strategies to cope with the stress signals [11, 16, 17]. To deal with the heat stress, a number of reports suggested the expression of the global molecular chaperones and other components (CspB and CspE in *Bacillus* spp. cells, GroEL and DnaK proteins in *Salmonella* spp., *rpoE*, *rpoS* and *rpoH* genes in *E. coli* and *Pseudomonas* spp.) to combat against the stress as well as to maintain the cellular homeostasis [5, 6, 13, 18–34].

Our earlier studies unraveled the influence of the temperature up-shift with the generation of oxidative stress retarding the amount of viable and culturable bacterial cells [5, 12, 13, 35]; spontaneous accumulation of the ROS not only at the beginning of the early stationary phase but also by the supplementation of hydrogen peroxide (H_2_O_2_) in the growing culture [2, 5, 32, 34] and the effect of different aeration speed on the cellular capability to produce the colony forming units on agar plates [33]. In all instances, the physiological response of *E. coli* SUBE01, *Pseudomonas* spp. SUBP01, *Salmonella* spp. SUBS01 and *Bacillus* spp. SUBB01 against oxidative stress was observed through their sustainability in retaining the culturable cells [5, 12, 13, 32–34, 36]. Besides, the information on the defense strategy of these bacteria especially those belonging to *Bacillus* spp. SUBB01 under the static condition was evidently noticed through their phenotypic behavior [34]. Along these lines of information, current study was conducted to further scrutinize the heat-shock response in *Bacillus* spp. SUBB01 under the shaking condition at 100 rpm in different culture media.

## Methods

### Demonstration of culturable *Bacillus* spp. SUBB01 under heat stress

Laboratory stock culture of *Bacillus* spp. SUBB01 and *E. coli* SUBE01 were used in this study. Experiments demonstrating the bacterial growth in terms of cell turbidity (optical density at 600 nm) and colony forming units (CFUs) were conducted as described earlier by Nur et al. [34]. Nutrient agar (NA), Luria–Bertani (LB) agar, nutrient broth (NB) and Luria–Bertani broth were used for the assay of culturability [13]. After 24 h of incubation on nutrient agar plates at 37 °C, one loopful of the bacterial culture was introduced into 5 ml nutrient broth followed by incubation at 37 °C for 4–6 h at 100 rpm (pre-culture). After adjusting the optical density of the pre-culture at 600 nm (OD_600_) to 0.1, 30 µL each was introduced into 2 different sets of 30 ml of nutrient broth and Luria–Bertani broth and incubated at 48, 50, 52, 53 and 54 °C at shaking condition (100 rpm). At the time points of 12 and 24 h, the cell growth was monitored by measuring OD_600_ and by counting the colony forming units (CFUs) [34]. All the experiments were conducted three times. Statistical analysis regarding bacterial growth was performed by determining P value through t test. Standard deviations for all data have been indicated by error bars.

Assessing cell viability was further confirmed by the spot tests [13, 32–34]. As described previously, each the culture suspension was serially diluted in 9 ml nutrient broth to obtain up to 10^−4^ fold dilution. From each dilution, an aliquot of 5 µl was dropped on to the nutrient agar and Luria–Bertani agar, dried off for 15 min, and finally the plates were incubated at 37 °C for 24 h. Spotting on the agar was accomplished at 24 h of growth.

### Demonstration of morphological changes

Simple staining (Crystal Violet, Hucker’s Solution) was conducted to assess the viability and the cellular morphology as previously done [32–34]. Spore staining (malachite green oxalate, safranin O) was conducted to differentiate the bacterial spores from vegetative cells following standard procedures [37]. An aliquot of 10 µl from the bacterial culture suspension was removed at 24 h of growth, and the cellular morphology, shape and organization were observed under the light microscope (Optima Biological Microscope G206, manufactured in Taiwan) at 1000× magnification [32].

### Preparation of organic and inorganic supplements

To prepare the extracellular fractions of bacteria (*E. coli* and *Bacillus* species), cells were grown separately in 6 different sets of Durham’s bottle containing 5 ml minimal broth, which were kept in a shaking water bath at 100 rpm for 24 h at 37 °C (optimum growth temperature) [13]. Subsequently, actively growing bacterial cells were centrifuged at 4000 rpm for 15 min, and the resulting pellets were collected. Afterward pellets were centrifuged at 4000 rpm for 15 min for 3 times with 10 % glycerol and 50 mM CaCl_2_, respectively. The resulting supernatants were collected and used as organic supplements to observe the possible retrieval of *E. coli* cell viability both at high temperature stress (45 °C) and around the critical temperature (47 °C). Subsequently, a mixture of 20 mM MgSO_4_ and 5 mM ethylene diamine tetraacetic acid (EDTA) were used as inorganic supplement to conduct a similar experiment [12].

## Results and discussions

### Growth retardation of *Bacillus* spp. SUBB01upon heat shock

**Fig. 1:**
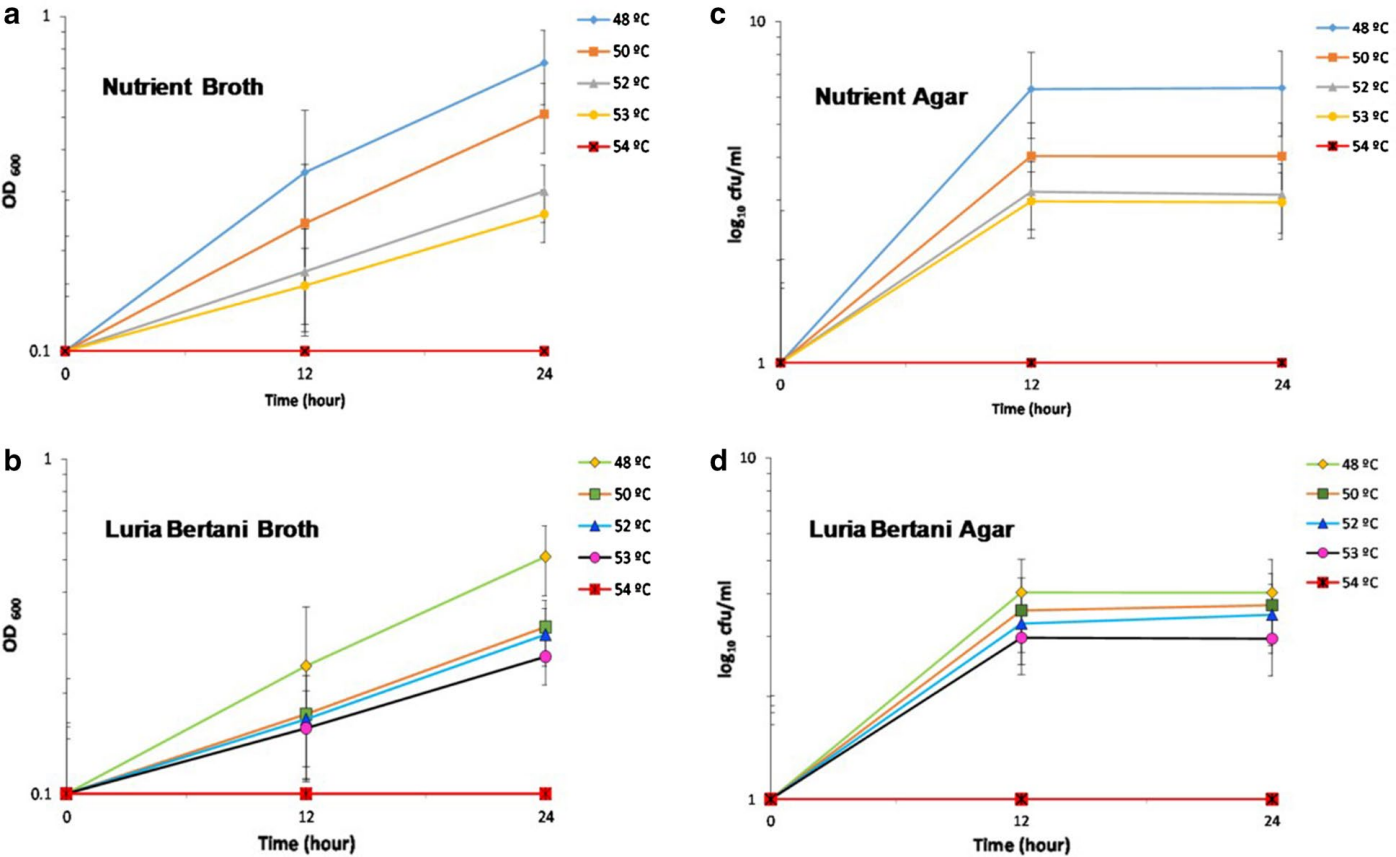
Growth retardation of *Bacillus* spp. SUBB01 at extended temperature. Assessment of growth of *Bacillus* spp. SUBB01 at 48, 50, 52, 53 and 54 °C in terms of cell turbidity (**a**, **b**) and colony forming unites (**c**, **d**) at 100 rpm. Cells were grown aerobically as stated in “Methods”. Aliquots were removed at 12 and 24 h for the assay of culturable cells. Retardation of growth was slightly observed in case of *Bacillus* spp. SUBB01 after 12–24 h in both LB broth and nutrient broth

As stated earlier, our former studies demonstrated the effective defense strategies of *Bacillus* spp. SUBB01 in response to the oxidative stress artificially created by the supplementation of 6 mM H_2_O_2_ into the growing culture at an aeration speed of 100 rpm, while the *E.coli* SUBE01 and *Pseudomonas* spp. SUBP01 were found to loose culurability under the same condition. Interestingly, as has been noticed in our earlier study, a decrease in the amount in the culturable cells of *Bacillus* spp. SUBB01 was observed when challenged with an increased concentration (21 mM) of H_2_O_2_ [34]. Apart from this stressed condition, the present study also employed a state of heat stress in the *Bacillus* cells since the increase in temperature is known to induce the accumulation of ROS inside the cells, which in turn largely accounts for losing cellular viability and culturability [35]. In this investigation, when the *Bacillus* spp. SUBB01 cells were grown at 48–53 °C, around 4-log reduction in cell turbidity (Fig. 1a, b) as well as in the generation of the colony forming units (CFUs) were observed (Fig. 1c, d) up to 24 h of incubation periods in both nutrient and Luria–Bertani (LB) agar and broth. Notably, even as high as at 53 °C, cells were noted to produce the CFUs up to 10^2^ CFU/ml. Interestingly, a complete elimination in both cell turbidity and colony forming units (CFUs) were observed when the cells were challenged at 54 °C, and hence indicating the critical temperature for *Bacillus* spp. SUBB01 to be at 53 °C (Fig. 1).

**Fig. 2:**
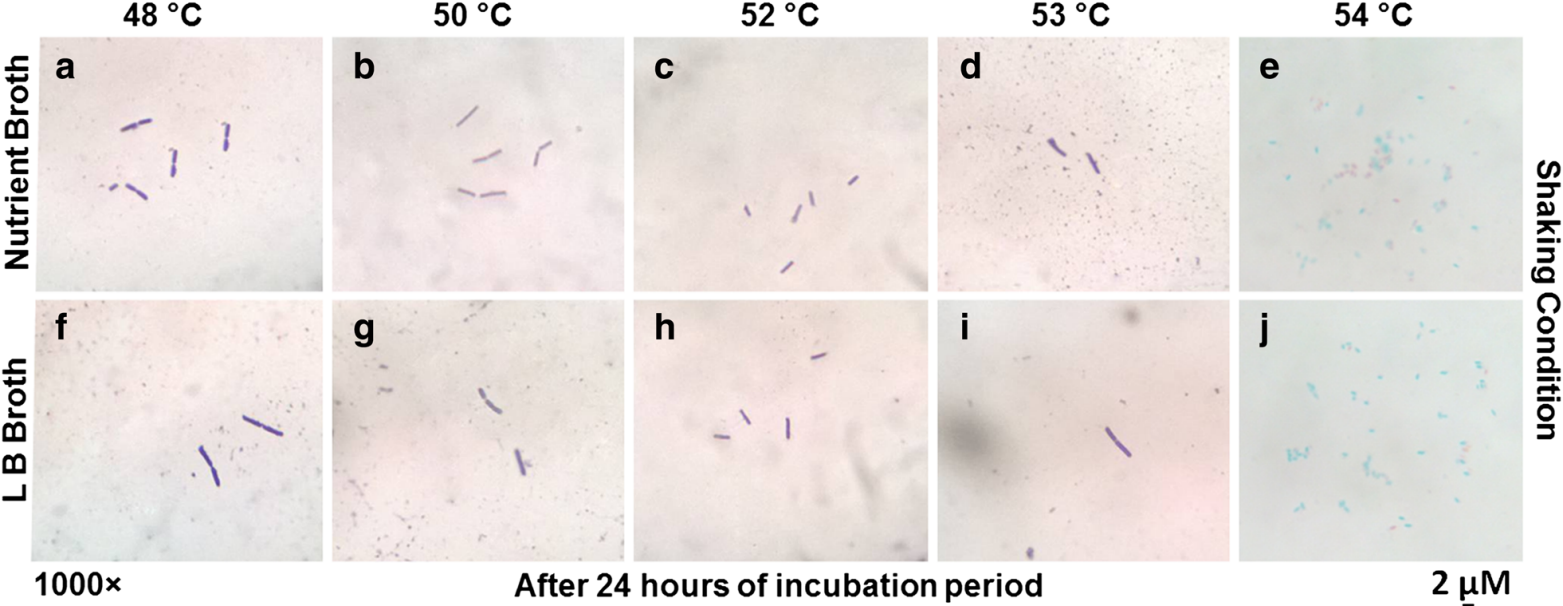
Cells were grown and aliquots were removed from the respective bacterial cultures for light microscopy as stated in the “Methods” section. However, no significant morphological or arrangement changes were observed regardless of heat shock at 48, 50, 52, 53 and 54 °C at 100 rpm (**a**–**j**)

Consistent with the results acquired in the growth related experiments, no morphological changes were observed under light microscope when cells were subjected to growth temperatures of 48, 50, 52, 53 °C in both LB and nutrient broth for up to 24 h of incubation (Fig. 2). However, sporulation was observed at 54 °C. Earlier studies showed that the stressosome signaling complex of *Bacillus* spp. is activated in response to several environmental stresses including the housekeeping σ factor (σ^A^) and alternative sigma factor (σ^B^) during the early or mid stationary phase, providing the protective adaption from environmental changes [16, 38–44]. Several other studies showed that in response to heat shock, σ^F^ has been found to be activated, which in turn may protect cells from heat shock through sporulation means [45]. Although such genetic analyses were not conducted in the present investigation; the results presented in our study clearly indicate that the cells lose their culturability at 54 °C, which indicates the dormant but viable state through sporulation.

### Confirmative demonstration of loss of culturability of *Bacillus* spp. SUBB01cells

**Fig. 3:**
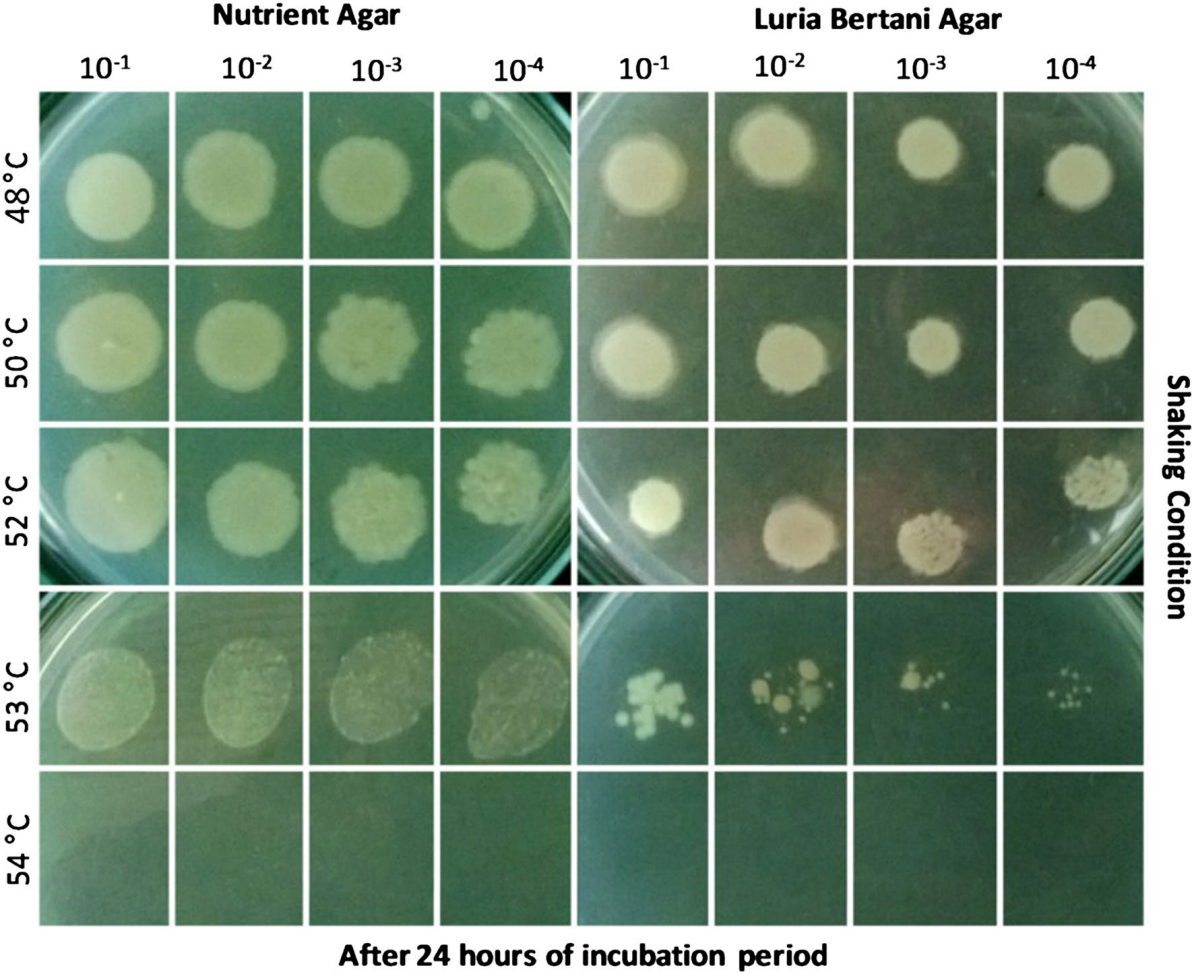
Confirmative demonstration of culturability and survival potential of *Bacillus* spp. SUBB01 cells upon heat shock. Cells were grown aerobically as stated earlier, and aliquots were removed at 24 h for spot tests. A progressing decline in bacterial growth was observed at 53 °C and the complete abolishment of cells was found at 54 °C after 24 h of incubation period in both nutrient agar media and Luria–Bertani agar media

The sharp decline in culturable cell population fraction (Fig. 1), as well as the presence of spores under light microscope due to extreme high temperature (imposed by 54 °C) in *Bacillus* spp. SUBB01 cells (Fig. 2), led us to further cross-check of the stressed physiology of the cells through the spot tests as corroborated earlier [13, 32–34]. Consistent to the growth experiments as shown in Fig. 1, a steady growth was observed at 48, 50 and 52 °C with minimal variation or growth reduction in both nutrient and LB agar (Fig. 3) and a relatively slower growth was observed when bacterial cells were grown at 53 °C on both nutrient and LB agar, whereas a complete growth cessation was observed at 54 °C (Fig. 3).

As stated earlier, our previous investigations on stress responses in *E. coli* SUBE01 showed the influence of the temperature up-shift resulting in oxidative stress [13] which further led us to investigate the stress response against external and internal oxidative stress stimuli within *Bacillus* spp. SUBB01, *E. coli* SUBE01, *Pseudomonas* spp. SUBP01 and *Salmonella* spp. SUBS01 [5, 12, 13, 32–34, 36]. Those studies clearly revealed that *E. coli* SUBE01 lost viability upon heat shock [13]. Moreover, the external and internal oxidative stresses in the early stationary phase of *E. coli* SUBE01 and *Pseudomonas* spp. SUBP01 culture were found to influence the formation of culturable cells; i.e., capable of forming colonies [2, 32–34]. With a succession of those work, the results presented in this study showed that *Bacillus* spp. SUBB01 is likely to exhibit the alteration in cellular homeostasis and culturability due to heat shock at 48–53 °C, under aeration (100 rpm) condition on different culture media. Notably, cells were found to lose culturablility completely at 54 °C, wherein spores were observed under light microscope (Fig. 2). Previously several studies reported that *Bacillus* cells exhibits six classes of heat shock genes upon environmental stress and the activation of the heat shock genes of *Bacillus* species especially depends on the commencement of specific temperature [45, 46].

### Demonstration of growth retrieval

**Fig. 4:**
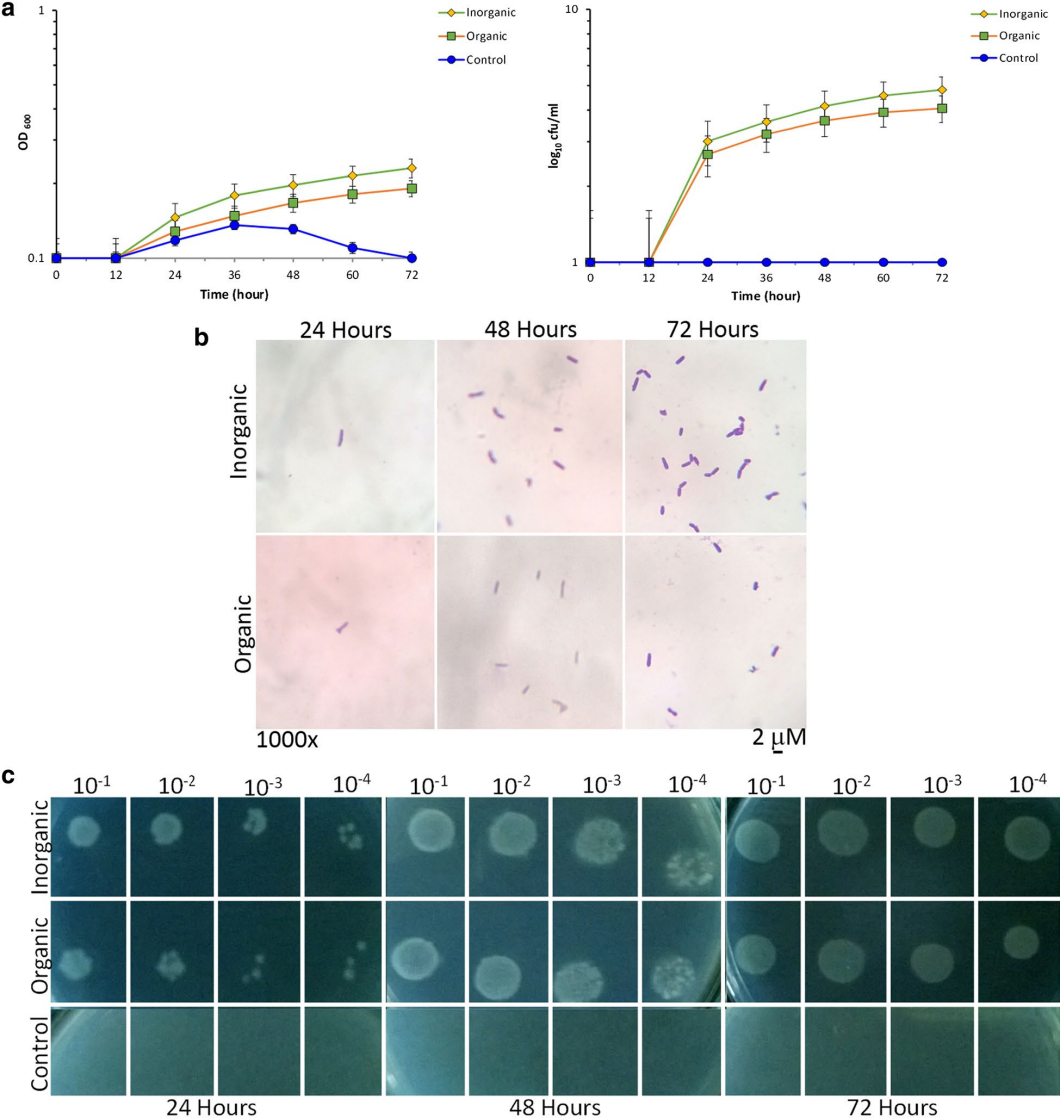
Growth revival assay of *E. coli* SUBE01 at critical growth temperature (45 °C). Effect of high temperature on growth of *E. coli*: **a** impact on cell turbidity and influence on the formation of CFUs, (**b**) and the demonstration of loss of cell culturability by means of spot test (**c**) upon supplementation with organic and inorganic supplement. Cells were grown in minimal at 45 °C up to 72 h and after 10 h of growth organic supplement (the extracellular fractions of *Bacillus* species) and inorganic supplement (20 mM MgSO_4_ with 5 mM EDTA) were added and untreated cells were referred as control. At every 12 h interval, cells were diluted and grown in minimal agar at 37 °C for 24 h. A growth revival was observed to be increased by approximately four logs for *E. coli* cells at different period of incubation. All experiments were carried out three times and the standard deviations for all data have been indicated by *error bars*

**Fig. 5:**
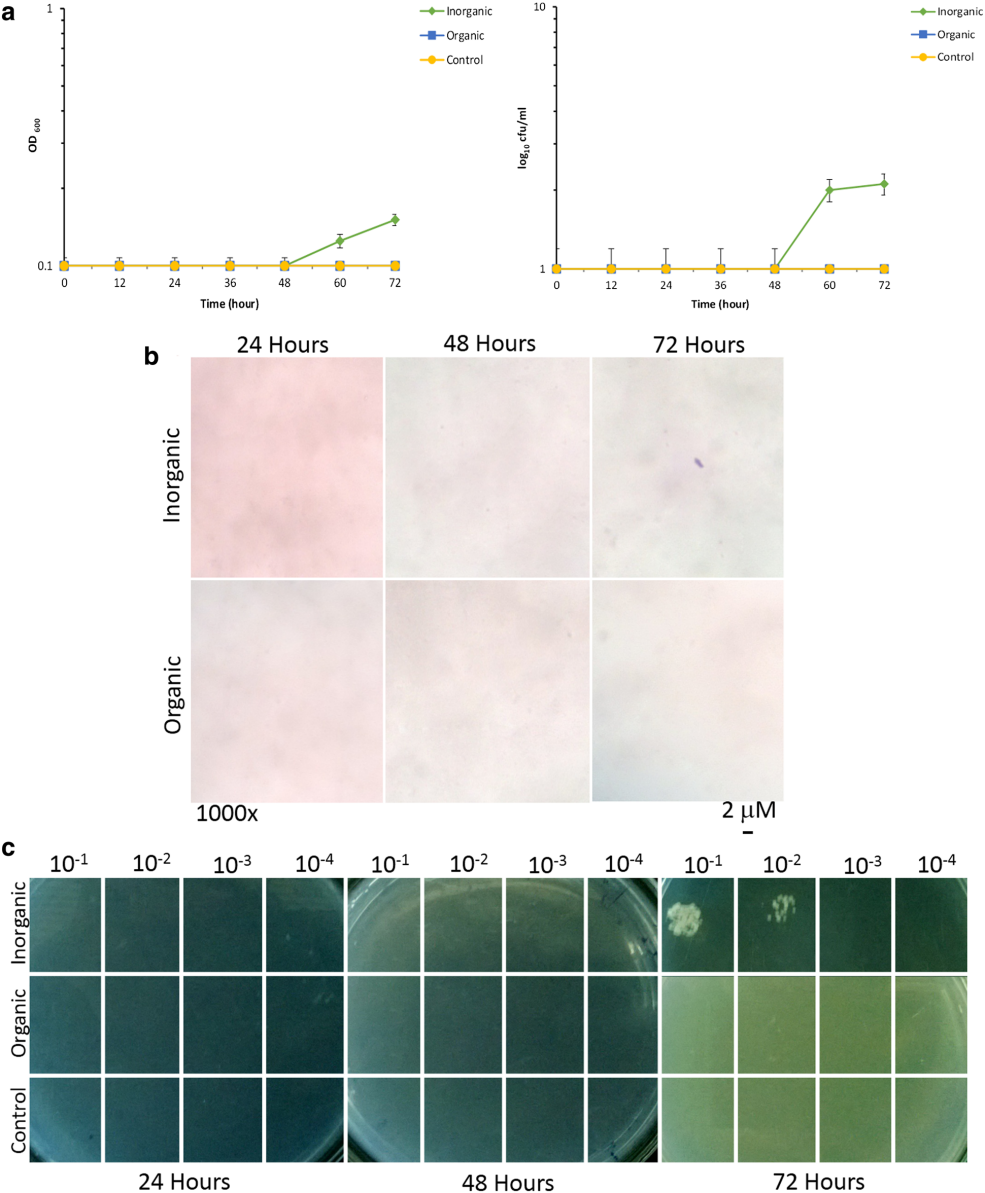
Growth revival assay of *E. coli* SUBE01 at above critical growth temperature (47 °C). Effect of high temperature on growth of *E. coli*: **a** impact on cell turbidity and influence on the formation of CFUs, **b** and the demonstration of loss of cell culturability by means of spot test **c** upon supplementation with organic and inorganic supplement. Cells were grown in minimal agar at 47 °C up to 72 h and after 10 h of growth organic supplement (the extracellular fractions of *Bacillus* species) and inorganic supplement (20 mM MgSO_4_ with 5 mM EDTA) were added and untreated cells were referred as control. At every 12 h interval, cells were diluted and grown in minimal agar at 37 °C for 24 h. A growth revival was observed at 47 °C (**c**, **d**), when cells were grown with inorganic supplement. All experiments were carried out three times and the standard deviations for all data have been indicated by *error bars*

**Fig. 6:**
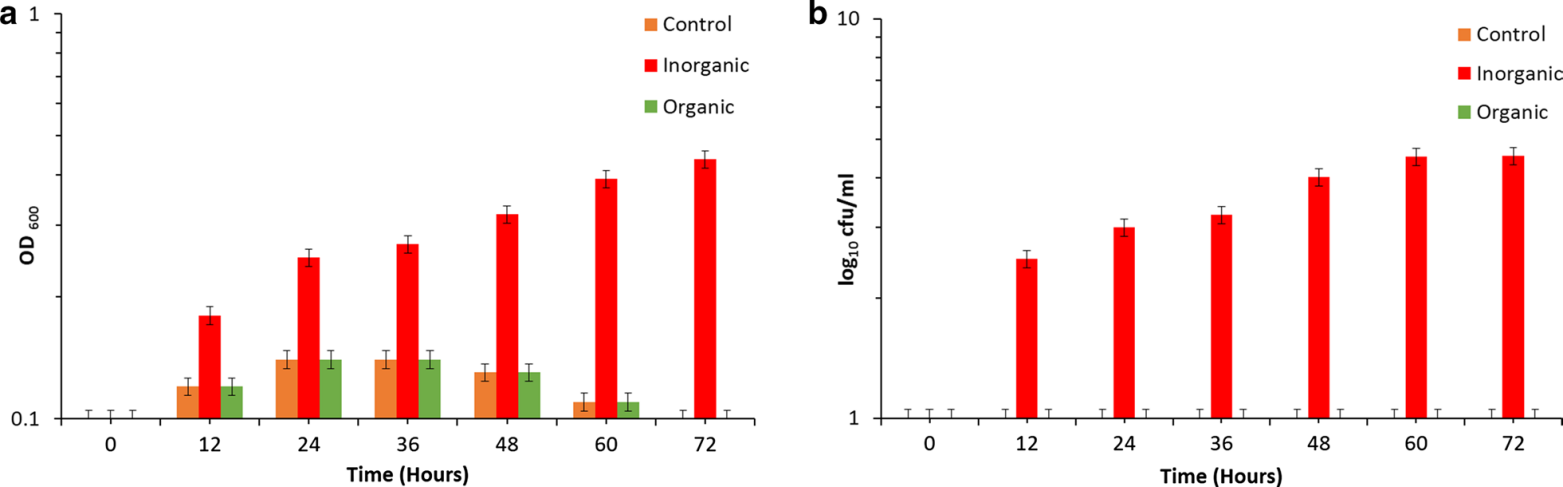
Impact of extracellular fractions of *E. coli* on cell culturability of *E. coli* at 45 °C. Effect of high temperature on growth of *E. coli* SUBE01: **a** impact on cell turbidity **b** and influence on the formation of CFUs, upon supplementation with the extracellular fractions of *E. coli* SUBE01 as organic supplement and 20 mM MgSO_4_ with 5 mM EDTA as inorganic supplement and untreated cells were referred as control. Cells were grown in minimal agar at 45 °C up to 72 h and after 10 h of growth organic supplement and inorganic supplement were added. At every 12 h interval, cells were diluted and grown in minimal agar at 37 °C for 24 h. No significant changes were observed at 45 °C (**a**, **b**), when cells were grown with the extracellular fractions of *E. coli* SUBE01. All experiments were carried out three times and the standard deviations for all data have been indicated by *error bars*

Another remarkable aspect was to evaluate the possible positive effect on the high temperature stressed *E. coli* SUBE01 growth at critical (45 °C) and above critical (47 °C) temperature [13] upon the supplementation of the extracellular fractions of *Bacillus* species as organic supplement which was further compared to the inorganic supplement into the cultures in the conditions as stated above (Figs. 4, 5). Previously it has been reported that Mg^2+^ and EDTA were capable of protecting the outer membrane from cell burst upon heat shock [12]. Concerning that factual report in the current investigation, when the stressed culture of *E. coli* SUBE01 cells were treated with the extracellular fractions of *Bacillus* spp. around their critical temperature in both minimal agar and broth media, cells were surprisingly found to retrieve their growth after certain incubation periods (Figs. 4, 5). Moreover, no significant changes were observed, when cells were grown with the extracellular fractions of *E. coli* SUBE01 compared to those treated with inorganic supplement as illustrated in Fig. 6.

As stated earlier, our previous studies unraveled the defensive strategies of various bacterial species against heat shock and oxidative stress [5, 12, 13, 32–36]. Recently the response within yeast cells against heat stress and osmotic shock has also been observed [47]. While the mechanisms of survival of *E. coli* cells have clearly been chalked out very recently [48], the retrieval of a heterogeneous *E. coli* population consisting of viable cells and defective cells (incapable of forming colonies on agar plates) by the *Bacillus* extracts as found in the current study is being reported for the first time so far to our knowledge. Such an experimental demonstration could be of significance to understand the cellular survival strategies mediated by different bacterial species.

## Conclusion

Despite the lack of molecular investigation as well as an apparent impression of descriptive nature of research stipulation, the data in the current study is quite consistent to the previous findings with the novel projection on the critical growth temperature of *Bacillus* strain. Moreover, the work clearly illustrated the phenotypic changes in the bacterial cell caused by the heat shock at the optimum speed of aeration which, unlike to that of *E. coli*, is relatively new in the field of heat shock response in *Bacillus* cells. Such preliminary findings could be worth incrementing the existing knowledge on bacterial cell biology and signal transduction. Finally, the observation of *E. coli* growth retrieval upon supplementation of the extracellular fractions from *Bacillus* spp. have been indeed interesting to ponder on the heat stress resistance mechanisms of *Bacillus* spp. However, further molecular studies on the genetic makeup of such stress responses as well as the growth retrieval mechanisms by means of exogenous organic factors (*Bacillus* extracts) would be of greater effectiveness.
